# Traveling Letter, No. VIII

**Published:** 1844-11

**Authors:** 


### TRAVELING LETTERS FROM THE SENIOR EDITOR.

NO. VIII.

CHICAGO, Ill., Sept. 24th, 1844.

*To my Colleagues:*

GENTLEMEN:—My last, finished at Joliet, only four days ago, has not reached you, before I begin another. But don't be alarmed, for I shall not close this under a week. The present and prospective importance of Chicago, resulting from its geopraphical position, seems to give it a claim to the attention of travelers of all sorts, beyond that of many other towns; and I am unwilling to leave it, without preparing a brief notice of such 'matters and things' concerning it, as may, by 'liberal construction,' be introduced into a medical journal, as part of a rambling letter from a rambling editor.

On approaching the town from the west, the traveler descends from the high, undulating prairies, over which he has been rapidly rolled along, towards a small river, called by the French pioneers, *Des Plaines,* which coming down from the north, parallel to the lake, bends to the south-west to unite, some miles below the crossings, with the Kaukakee, which flows from the south-east. The junction of these streams, forms the Illinois, as the Allegheny and Monongahela unite to form the Ohio. For a few miles before arriving at the Des Plaines, one finds himself traversing a level prairie, elevated a few feet above the surface of the little river, and after fording it, he continues his journey over the same kind of surface for ten or twelve miles, when he reaches the new city, whose streets rise but six or eight feet above the surface of the Lake. This extensive plain, which has suggested the expressive name of the river which flows through it, is not without some declivity both towards the river and the lake; but, nevertheless is so flat, that the rank vegetation which it supports, prevents the rain from flowing off, and thus creates a permanently wet, if not marshy surface, which, however, a little ditching would render sufficiently dry for cultivation. As yet this has been done but to a very limited extent, and one is surprised to see, for ten miles before him. the spires, and masts of a beautiful and thriving commercial town, while he is still in the midst of the 'pontine marshes.' But he need not dread an algid or soporose intermittent, for even the simple, is almost unknown both in Chicago and its neighborhood.

The harbor of the town consists of the estuary of a small river called the Chicago, which consists of a north and south fork, meeting within the limits of the town. Between the latter, and the Des Plaines river, which are nearly on the same level, there is such communication, in times of great rains, that canoes have passed from one to the other. Indeed, when the lake was only twenty feet higher than at present, it covered the plain which I have described, and a great river flowed from it down the Valley of the Illinois, to disembogue in the Gulf of Mexico. In the States of Pennsylvania, Ohio, Indiana, and Illinois, there is no other spot where the watershed between the lakes and the rivers of the South, is so depressed as at this point; hence it was the last to transmit the waters of the lake to the Mississippi, and of the whole, it most favors a navigable communication between the North and South. The canal, partly excavated by the State of Illinois, passes through this depression, and when finished will be the best water highway between them that can ever be made, except, perhaps, that between the Wisconsin and Fox river, which discharges its waters into the head of Green Bay—a route that will forever possess the distinction of having been traversed by Marquette and Joliet, in the voyage which led to the discovery of the Mississippi.

Chicago is the head of navigation on Lake Michigan; the port of embarkation for travelers from the south-west, who would reach New York or Boston by Mackinac and Detroit; the place of debarkation for travelers going in the opposite direction, and for emigrants from the northern States to Illinois, Iowa, and Missouri. You will not be surprised, then, to learn, that in 13 years, it has risen from a few families, to a population of nearly 10,000; that it revels in high anticipations; and cherishes quite as much ambition as any other precocious American city.

Now, lest some of our readers, desirous of changing their location, should resolve to put off for Chicago, I feel it my duty to tell them, that she is already abundantly furnished with physicians. Of the exact number I cannot speak, but the *signs* of an adequte supply are conspicuous; and arrangements have been made for keeping it up, by home manufacture. Of this I gave you intimation in my last, expressing some disapprobation of the proposal to manufacture them here, and in some other places, at so small a cost. In the four days which have elapsed since the date of that letter, my opinions have undergone no change, and of course I still think, that as a cutler, who might advertise to make surgical instruments at half price, would not be patronized by our operators, so, surgeons made at half-price, should not, *a priori*, be regarded by the people, as strong and acute. Among the instruments made by the manufacturer of 'cheap goods,' there might now and then be one of excellent temper, and among the alumni of our cheap schools there may be found some of excellent professional temperament, but neither the shops nor the schools should be judged by these exceptions. Justice, however, to Dr. Brainard, the enlightened founder of the 'Rush Medical College' of this place, requires me to state, that he himself, in the abstract, does not approve of cheapening medical instruction; but says he was driven into it by the example of the schools in this latitude, from Geneva, in New York, to Fox River, in Illinois, embracing the inermediate establishments of Wil loughby, Cleveland, Laport, and Jacksonville. He and his colleagues, indeed, have it in view, in due time, to advance the price of their tickets. He does not anticipate a very rapid growth of his establishment, and would not, in the infancy of the country, have moved in its organization, but that several towns, which he justly regarded as too small and sequestered, had been made the sites of such institutions. I must confess that this view of the matter is plausible; and that if the patronage which would be distributed among the towns of Laporte, Jacksonville, and St. Charles, can be concentrated on Chicago, it will be for the benefit of the profession and society at large. Indeed, it must, I think, be admitted, that the towns just named, together with Willoughby, in the State of Ohio, are not places where flourishing medical colleges can be built. West of Pennsylvania and New York, leaving out of view the towns on or near the Ohio, the three points favoring and requiring such seminaries, are Saint Louis, Chicago, and Cleveland. The first is connected with the Lower and Upper Mississippi and with Western Illinois; the second has connection with Northern Indiana, middle and Northern Illinois, Western and Northern Michigan, Wisconsin and Iowa; the third may look to Michigan, North-eastern Indiana, Northern Ohio, North-western Pennsylvania, and Upper Canada; within which various States and Territories, there will, in another age, be a dense population, supplying students enough for three schools, but not for the *eight* which have already announced themselves. Each of the towns which I have mentioned, will have population enough to give business to a faculty of teachers, and subjects for the practical anatomist; each will have a hospital, where clinical medicine and morbid anatomy may be taught; and each will erect such literary and scientific associations, as will favor research and ambitious study, in both professors and pupils.

The first annual catalogue of the school of this place, just published, embraces 22 students, which is rather more than the first class in Transylvania University, or the Medical College of Ohio, and beyond what might have been expected on a spot, which 15 years ago was but a military post and the site of an Indian Agency.

I have made a visit to the edifice of the school—(to be finished in time for the ensuing course of lectures)—which will be commodious and respectable. The money with which the Trustees are building it, has been cheerfully contributed by the citizens of Chicago. The faculty have also commenced a *monthly*, under the title of the "Illinois Medical and Surgical Journal," of which Prof. Blaney is the Editor. Each number contains 16 large and well-printed pages the contents of which I have not had time to study, but observe a large proportion of original matter. Thus Chicago is fairly in the field, and her nearest rival will be St. Louis. I hope they will set an example to their seniors, of honorable bearing in the *certamen gloriæ.*

WISCONSIN TERRITORY,
*Milwaukee*, Sept. 29.

On the Western shore of Lake Michigan, there are four flourishing towns—Chicago, of which I have just spoken, in the State of Illinois, and South Port, Racine, and that in which I am now writing, in the Territory of Wisconsin. At South Port I did not stop, but in Racine I spent two days, and met with several intelligent physicians. I found here, as further South, that the last spring and summer, had been unusually wet, but the prevalence of autumnal fever not great. Of all the towns in East Wisconsin, that where I now am is the largest. Within its limits there is a great deal of lacustrine marsh, and yet it suffers but little from the fevers of autumn. This exemption is referable to two causes—first, the daily fluctuations of the Lake, by which the marsh is drenched; and second, the high latitude, 43°, giving a summer of too low a temperature to favor the development of malaria. At Green Bay, in lat. 44° 40′, I found, when I visited it in 1842, that intermittent and remittent fevers, are almost unknown, although many conditions requisite to their production are present. The climate overrules them. If I descend from these generalizations, to a few miscellaneous details, I hope you will not regard them as beneath the dignity of the vehicle in which I would send them abroad. First; the emigrant physician to these parts, should provide himself with Mr. Lapham's "*Geographical and Topographical description of Wisconsin,*" a scientific and instructive little volume, just published. When the canal at Louisville was excavated, Mr. L. was a youth who carried the leveler's rod; he is now the natural and statistical historian of this distant territory, and has given a very good account of its geography, geology, and botany, with notices of its climate and diseases, not unworthy the attention of the medical man, who may seek a residence here. Second; Milwaukie is destined to be a pretty town, from the *natural* hue, so to speak, of its bricks, which, when burnt, are of a light yellow, cream color; pleasanter to the eye, than the finest shades of Louisville or Cincinnati house painting, and apparently as hard and durable as bricks made of clay which burns red. Third; the physicians of Wisconsin and Northern Illinois, have a class of patients which we never see on the banks of the Ohio—immigrants from Norway; who in their anatomy and physiology, resemble ourselves more, I think, than the Germans do. They are very fair, with blue eyes and sandy hair. Fourth; but the glory of Wisconsin is not in its historians, its bricks, or its Norwegians, but its potatoes; on which a young doctor may pass his probation, with two cents a day, and come out of it in much better case, than the bear leaves his hybernaculum. These *meshannocks*, (I know not the classical orthography) would sell for a profit in the South, and the physician who might receive them in exchange for his boluses, would find it to his interest to ship them in that direction.

With this important annunciation, I will lay down my pen, and prepare for my journey across the Territory to the Mississippi river.

MADISON, October 3d.

Two days of stage travel has brought me to the capital of the Territory, where I propose to close my epistle. Its distance is nearly 100 miles from Milwaukie, its latitude a few minutes further north, being about 15′ north of 43°. It is beautifully situated, between the two upper of a chain of four romantic little lakes, which discharge their surplus waters into Rock river. The surrounding country, although thinly peopled, is fertile and fair in its aspects; the seat of Government it is expected will continue here after the Territory has passed through its chrysalis state; and the proportional number of physicians, I am told, is not great. This, therefore, would seem to be not an unfavorable spot for one or two good immigrant doctors, especially as the lakes abound in the finny races; and the young practitioner might add fish to his potatoes,—a diet highly favorable to sound health, hard study, and patient waiting.

I am now on the confines of the 'Mineral Regions,' and shall make it the subject of my next.

Respectfully,

Your friend and colleague,

